# Supplementary material for: Use of State Sequence Analysis in Pharmacoepidemiology: A Tutorial
Source: Int J Environ Res Public Health. 2021 Dec 20;18(24):13398. doi: 10.3390/ijerph182413398 (PMC8705850; doi:10.3390/ijerph182413398)
Supplement: Supplementary file 1 [file ijerph-18-13398-s001.zip › ijerph-1451606-supplementary.pdf]

Supplementary Materials: Use of State Sequence Analysis in Pharmacoepidemiology: A Tutorial

Jacopo Vanoli [1,2], Consuelo Rubina Nava [3,*], Chiara Airoldi [4], Andrealuna Ucciero [4], Virginio Salvi [5] and Francesco Barone-Adesi [5]

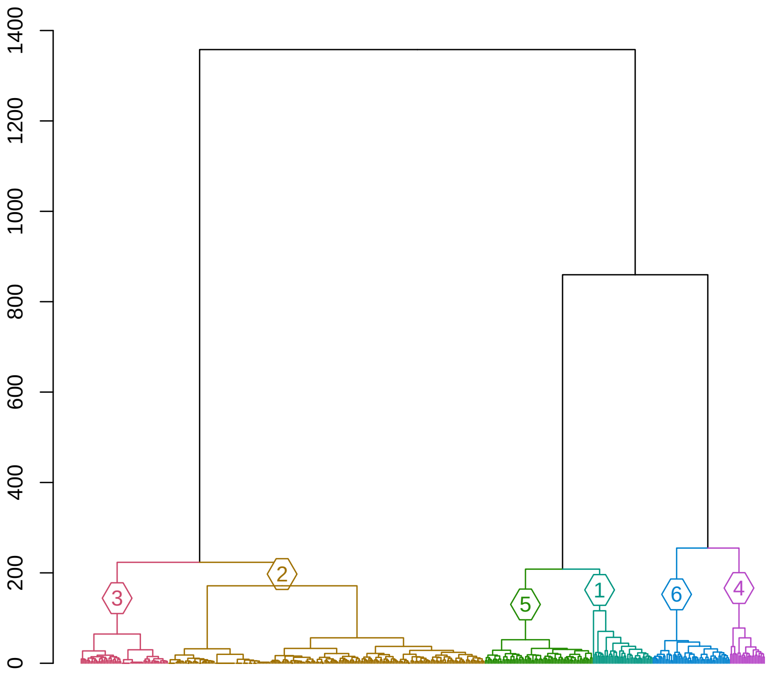

Figure S1. Dendrogram and identification of the six clusters.

File S1. R Code

```
###############
## LOAD LIBRARY
###############
library(openxlsx)
require(TraMineR)
require(sas7bdat)
require(TraMineRextras)
library(cluster)

###############
## LOAD FUNCTIONS
###############
# The "su" function compare two sequences from different  datasets
su<-function(x, y) {
  ok<-vector()
  for (i in 1:nrow(x)) {
    ok[i]<-seqcomp(x[i,],y[i,])
  }
  return(ok)
}

# The "funz" function generates a plot of the granularity.
# In particular, the~arguments are:
# lui = dataset that need a granularity reduction
# n = maximum number of granularities
# The output is a plot where the granularity values appear
```

```
# on the X axis while on Y axis the percentage of identical
# sequences between the original dataset
# and those with modified~granularity.

funz<- function(lui,n) {
  odd<-vector()
  do<-vector()
  dato<-seqdef(lui)
  tot<-seqdss(dato)

  for (i in seq(2, n, 1)) {
    if(i==19) next
    odd[i]<-i
    time<-bibla(seqdata=dato, tspan =i , method = "mostfreq")
    timo<-seqdss(time)
    k<-as.data.frame(table(su(timo, tot)))
    do[i]<-k$Freq[2]/nrow(dato)

  }

  return(plot(odd,do, type="o",pch=16,
  xlab="days␣of␣granularity",
  ylab= "proportions␣of␣equal␣sequences"))

}


# The "bibla" function is a modification of seqgranulairty()
# one due to some possible empirical issues of the~latter.

bibla<-function (seqdata, tspan = 3, method = "last")
{
  metlist <- c("first", "last", "mostfreq")
  if (!method %in% metlist) {
    stop("␣[!]␣method␣must␣be␣one␣of:␣",
    paste(metlist, collapse = "␣"),
         call. = FALSE)
  }
  n <- nrow(seqdata)
  lgth <- max(seqlength(seqdata))
  new.lgth <- ceiling(lgth/tspan)
  new.lgth.f <- floor(lgth/tspan)
  newseq <- seqdata[, 1:new.lgth]
  cnames <- names(seqdata)
  newcnames <- cnames[seq(from = 1, to~= lgth, by~= tspan)]
  prev <- 0
  if (method == "first")
    prev <- tspan - 1
  for (i in 1:new.lgth.f) {
    newseq[, i] <- seqdata[, tspan * i - prev]
  }
  if (method == "mostfreq") {
    for (i in 1:new.lgth.f) {
      st.freq <- suppressMessages(seqistatd(seqdata[, (tspan *
    (i - 1) + 1):(tspan * i)]))
      newseq[, i] <- apply(st.freq, 1, function(x) {
        names(which.max(x))
      })
    }
  }
  if (new.lgth > new.lgth.f) {
    if (method == "first") {
      newseq[, new.lgth] <- seqdata[, tspan * new.lgth.f + 1]
    }
    else if (method == "mostfreq") {
      st.freq <- suppressMessages(seqistatd(seqdata[, (tspan *
      new.lgth.f):lgth]))
      newseq[, new.lgth] <- apply(st.freq, 1, function(x) {
        names(which.max(x))
```

```
      })
    }
    else {
      newseq[, new.lgth] <- seqdata[, lgth]
    }
    newcnames[new.lgth] <- cnames[lgth]
  }
  colnames(newseq) <- newcnames
  attr(newseq, "xtstep") <- ceiling(attr(seqdata, "xtstep")/tspan)
  return(newseq)
}

###################
### LOAD DATA #####
###################

data_sts2<-read.table("Data_Example.txt")

###############
## WEEKLY DATA
###############

# Select granularity here is equal to 7 and considering only the first year
first_y<-seqdef(data_sts2[,1:364])
week<-seqdef(bibla(first_y, tspan = 7,
method = "mostfreq"))

# contruct an object collecting also in the last three columns
# the number of weeks given the states  1=weak, 2=strong, 3=pause
year<-as.data.frame(cbind(id_preserve1,seqici(week),
      seqient(week), seqST(week),
      seqistatd(week)))
colnames(year)[1]<-"id"
colnames(year)[2]<-"Complexity"


# select and drop subject that start with a pause (3)
id_rm<-which(week$y18994==3)
week_nopause<-week[-id_rm,]
year_nopause<-as.data.frame(cbind(id_preserve1[-id_rm,], seqici(week_nopause),
      seqient(week_nopause),seqST(week_nopause), seqistatd(week_nopause)))
colnames(year_nopause)[1]<-"id"
colnames(year_nopause)[2]<-"Complexity"


###################
## CLUSTER ANALYSIS
###################
# cluster analysis based on week_nopause
dist_r<-seqdist(week_nopause,method = "LCS", full.matrix = F)
singleclust_r <- hclust(dist_r, method = "ward.D")

# evaluate the dendrogram structure
plot(singleclust, xlab="observations", ylab="height", main="")

# here we extract 6 clusters
cluster6<-cutree(singleclust,k = 6)

# dendrogram plot
pdf("dendro_r.pdf")
plot(singleclust_r, xlab="observations", ylab="height", main="",
     hang=-1, labels=FALSE)
rect.hclust(singleclust_r, k=6, border=c(2,3,4,5,6,7))
dev.off()

# assign cluster id to each sequence
week_nopause$cluster6<-cutree(singleclust_r,k = 6)

# count how many Weak, Strong and Pause for each sequence
```

```
num1_r<-c()
num2_r<-c()
num3_r<-c()
for (i in 1:dim(week_nopause)[1]){
  num1_r<-c(num1_r,length(which(c(week_nopause[i,])==1)))
  num2_r<-c(num2_r,length(which(c(week_nopause[i,])==2)))
  num3_r<-c(num3_r,length(which(c(week_nopause[i,])==3)))
}

# assign them to the completa dataset
week_nopause$num1<-num1_r
week_nopause$num2<-num2_r
week_nopause$num3<-num3_r

# select sequences for each cluster
ind6.1_r<-which(week_nopause$cluster6==1)
set6.1_r<-week_nopause[ind6.1_r,]
ind6.2_r<-which(week_nopause$cluster6==2)
set6.2_r<-week_nopause[ind6.2_r,]
ind6.3_r<-which(week_nopause$cluster6==3)
set6.3_r<-week_nopause[ind6.3_r,]
ind6.4_r<-which(week_nopause$cluster6==4)
set6.4_r<-week_nopause[ind6.4_r,]
ind6.5_r<-which(week_nopause$cluster6==5)
set6.5_r<-week_nopause[ind6.5_r,]
ind6.6_r<-which(week_nopause$cluster6==6)
set6.6_r<-week_nopause[ind6.6_r,]

# compute mean value for each cluster
mean_state6_r<-rbind(
                    round(colMeans(set6.1_r[,c(54:56)]),2),
                    round(colMeans(set6.2_r[,c(54:56)]),2),
                    round(colMeans(set6.3_r[,c(54:56)]),2),
                    round(colMeans(set6.4_r[,c(54:56)]),2),
                    round(colMeans(set6.5_r[,c(54:56)]),2),
                    round(colMeans(set6.6_r[,c(54:56)]),2))

# integrate all available information in a unique object
week_nopause$id<-unlist(id_preserve1[-id_rm,])
ind_orig_r<-c()
prescription_r<-c()
j=1
for (i in 1:length(week_nopause$id)){
  ind_id<-week_nopause$id[i]
  ind_orig_full<-which(data_orig$id==ind_id)
  ind_orig_r[j]<-ind_orig_full[1]
  prescription_r[j]<-length(ind_orig_full)
  j=j+1
}

week_nopause$ind_orig<-ind_orig_r
week_nopause$npres<-prescription_r

week_final<-cbind(
     week_nopause[,-c(54:56,58:59)],
     year_nopause[,-1])
# rename columns
names(week_final)[1:52]<-1:52
names(week_final)[58:60]<-c("n_weeks_weak",
     "n_weeks_strong","n_weeks_notreat")

##############
# COMPLEXITY INDEXES
##############
# Elzinga (turbolence)
elzinga<-seqST(week_final)

# Entropy
entro<-seqient(week_final, norm = FALSE)
```

```
##############
# PLOTS
##############

# Cluster plot
pdf("plot_cluster6.pdf", height = 5, width = 10)
week2<-week_nopause[ind6.1_r,1:52]
names(week2)<-1:52
seqplot(week2, type="I", with.legend = FALSE, xlab="Weeks",
ylab="", main="Cluster1(41)", cpal=c("gold","firebrick2","gray80"))
week2<-week_nopause[ind6.2_r,1:52]
names(week2)<-1:52
seqplot(week2, type="I", with.legend = FALSE, xlab="Weeks",
ylab="", main="Cluster2(217)", cpal=c("gold","firebrick2","gray80"))
week2<-week_nopause[ind6.3_r,1:52]
names(week2)<-1:52
seqplot(week2, type="I", with.legend = FALSE, xlab="Weeks",
ylab="", main="Cluster3(60)", cpal=c("gold","firebrick2","gray80"))
week2<-week_nopause[ind6.4_r,1:52]
names(week2)<-1:52
seqplot(week2, type="I", with.legend = FALSE, xlab="Weeks",
ylab="", main="Cluster4(24)", cpal=c("gold","firebrick2","gray80"))
week2<-week_nopause[ind6.5_r,1:52]
names(week2)<-1:52
seqplot(week2, type="I", with.legend = FALSE, xlab="Weeks",
ylab="", main="Cluster5(74)", cpal=c("gold","firebrick2","gray80"))
week2<-week_nopause[ind6.6_r,1:52]
names(week2)<-1:52
seqplot(week2, type="I", with.legend = FALSE, xlab="Weeks",
ylab="", main="Cluster6(53)", cpal=c("gold","firebrick2","gray80"))
dev.off()

# state distribution plot
pdf("plot_state_distrib.pdf", height = 7, width = 10)
week2<-week_nopause[,1:52]
names(week2)<-1:52
seqdplot(week2, border = NA, xlab="Weeks",
        ylab="Proportion of patient", cpal=c("gold","firebrick2","gray80"),
        ltext=c("weak opiod","strong opioid","no treatment"))
dev.off()

# state distribution plot by clusters
pdf("plot_state_distrib_bycluster.pdf", height = 7, width = 10)
week2<-week_nopause[ind6.1_r,1:52]
names(week2)<-1:52
seqdplot(week2, border = NA, xlab="Weeks",
        ylab="Proportion of patient", main="Cluster1",
        cpal=c("gold","firebrick2","gray80"),
        ltext=c("weak opiod","strong opioid","no treatment"))
week2<-week_nopause[ind6.2_r,1:52]
names(week2)<-1:52
seqdplot(week2, border = NA, xlab="Weeks",
        ylab="Proportion of patient", main="Cluster2",
        cpal=c("gold","firebrick2","gray80"),
        ltext=c("weak opiod","strong opioid","no treatment"))
week2<-week_nopause[ind6.3_r,1:52]
names(week2)<-1:52
seqdplot(week2, border = NA, xlab="Weeks",
        ylab="Proportion of patient", main="Cluster3",
        cpal=c("gold","firebrick2","gray80"),
        ltext=c("weak opiod","strong opioid","no treatment"))
week2<-week_nopause[ind6.4_r,1:52]
names(week2)<-1:52
seqdplot(week2, border = NA, xlab="Weeks",
        ylab="Proportion of patient", main="Cluster4",
        cpal=c("gold","firebrick2","gray80"),
        ltext=c("weak opiod","strong opioid","no treatment"))
```

```
week2<-week_nopause[ind6.5_r,1:52]
names(week2)<-1:52
seqdplot(week2, border = NA, xlab="Weeks",
         ylab="Proportion␣of␣patient", main="Cluster5",
         cpal=c("gold","firebrick2","gray80"),
         ltext=c("weak␣opiod","strong␣opioid","no␣treatment"))
week2<-week_nopause[ind6.6_r,1:52]
names(week2)<-1:52
seqdplot(week2, border = NA, xlab="Weeks",
         ylab="Proportion␣of␣patient", main="Cluster6",
         cpal=c("gold","firebrick2","gray80"),
         ltext=c("weak␣opiod","strong␣opioid","no␣treatment"))

dev.off()
```
